# Step-by-Step Cadaveric Dissection of the Carotid Region: Anatomical Landmarks, Surgical Exposure, and Clinical Applications

**DOI:** 10.7759/cureus.115930

**Published:** 2026-09-08

**Authors:** Rhyan Ouaddane Alami, Abdesslam Bouassria, Soufiane Mellas, Mustapha Elkouache, Khalid Chakour

**Affiliations:** 1 Medicine, Sidi Mohamed Ben Abdellah University, Fez, MAR; 2 Urology, Hassan II University Hospital, Fez, MAR; 3 Laboratory of Anatomy and Surgery, Sidi Mohamed Ben Abdellah University, Fez, MAR

**Keywords:** cadaveric dissection, carotid bifurcation, carotid region, carotid sheath, farabeuf's triangle, hypoglossal nerve, surgical anatomy

## Abstract

The carotid region represents critical neurovascular spaces of the anterolateral neck, where a precise three-dimensional understanding of vascular, neural, and fascial relationships is essential for safe cervical surgery. This article describes a standardized, step-by-step cadaveric dissection protocol linking consistent anatomical landmarks to their surgical relevance. Unilateral dissections were performed on a formalin-preserved adult human cadaver using standard gross anatomical techniques. Specimens were dissected from superficial to deep layers according to a six-step sequence based on classical anatomical methodology, The protocol outlines: surface landmark identification; subplatysmal dissection and sternocleidomastoid muscle mobilization; exposure of the internal jugular vein and Farabeuf's triangle; management of venous tributaries and carotid sheath opening; carotid bifurcation exposure; and mapping of adjacent neural structures including the hypoglossal and vagus nerves, ansa cervicalis, and carotid body. This standardized cadaveric framework provides a structured approach to mastering the complex three-dimensional anatomy of the carotid region, serving as a practical educational reference for vascular, head and neck, and cervical spine surgery.

## Introduction

The carotid region (regio carotica) is a quadrilateral anatomical space of the anterolateral neck, obliquely oriented superiorly and posteriorly. It is bounded anteriorly and posteriorly by the borders of the sternocleidomastoid muscle (SCM), superiorly by the posterior belly of the digastric muscle, inferiorly by the clavicle and jugular notch, and deeply by the cervical spine and visceral axis. Within these boundaries lie critical neurovascular structures, including the common, internal, and external carotid arteries, internal jugular vein, vagus nerve, hypoglossal nerve, and branches of the cervical plexus [1,2].

Given its intimate neurovascular relationships, safe exposure of this region is paramount during carotid endarterectomy, selective neck dissections, carotid body tumor resections, and anterior cervical approaches. However, anatomical variations-such as the level of carotid bifurcation, external carotid branching, and venous drainage patterns-frequently modify the surgical field.

We thank the reviewer for this very positive assessment of our work. We are pleased that the reviewer found our conclusions to be well-supported by both the structured text and the sequential photographic documentation, confirming the pedagogical value and overall clarity of our cadaveric technical report.

## Technical report

Materials and methods

Anatomical Material

Dissections were performed on one formalin-preserved adult human cadaver (right neck side) at the Department of Anatomy, Faculty of Medicine and Pharmacy of Fez. Specimens with previous neck surgery, major local deformity, or significant pathological alteration of the cervical region were excluded. Unilateral dissections were performed. All anatomical specimens in our laboratory were derived from body donation programs conducted in accordance with institutional ethical guidelines and national legal frameworks governing medical education and research. 

Because the primary aim of this report is to provide a reproducible, sequential educational roadmap for surgical trainees rather than a morphometric population analysis, quantitative measurements were not collected.

To ensure high procedural accuracy and anatomical fidelity, the dissection was conducted by an expert team comprising senior academic anatomists with over 10 years of experience in cadaveric dissection and surgical training.

Dissection Technique

The cadaver is positioned supine with slight cervical extension and rotation of the head to the contralateral side, reproducing a conventional surgical position. Standard anatomical instruments are used, including scalpel blades, Metzenbaum and iris scissors, toothed and non-toothed forceps, groove directors, and Farabeuf retractors. The quadrilateral carotid region and its deeper carotid triangle are dissected systematically from superficial to deep structures according to a six-step protocol based on classical anatomical methodology.

Results

Step-by-Step Dissection Protocol

Step one - surface landmarks and initial incision: Dissection begins by identifying the superficial boundaries of the overall quadrilateral carotid region via surface palpation. The body and margins of the SCM, the mastoid process cranially, and the upper border of the clavicle and jugular notch caudally. Skin flaps are reflected laterally following a three-line incision: proximally 1 cm above the inferior border of the mandible extending toward the mastoid process, vertically down the midline to 2 cm below the jugular notch, and distally along the clavicle. Subplatysmal dissection is subsequently performed, preserving the platysma fibers, external jugular vein, and great auricular nerve on the superficial surface of the SCM to establish clear spatial orientation prior to entering deeper cervical fascial layers (Figures 1, 2).

**Figure 1:**
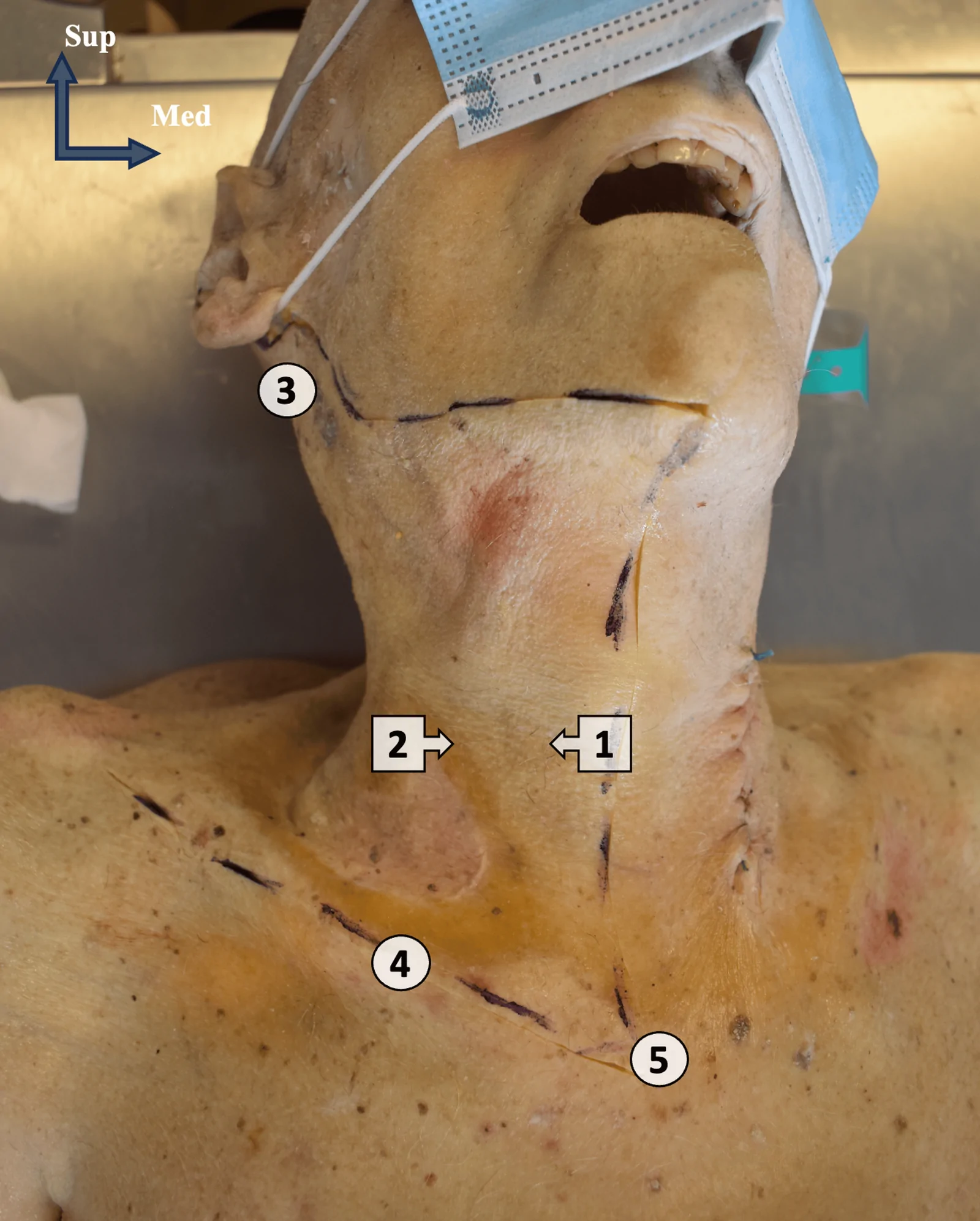
Anterolateral view of the right neck side illustrating the superficial anatomical limits prior to incision. Anterior border of SCM (1), Posterior border of SCM (2), Mastoid process (3), Clavicle (4), Jugular notch (5).

**Figure 2:**
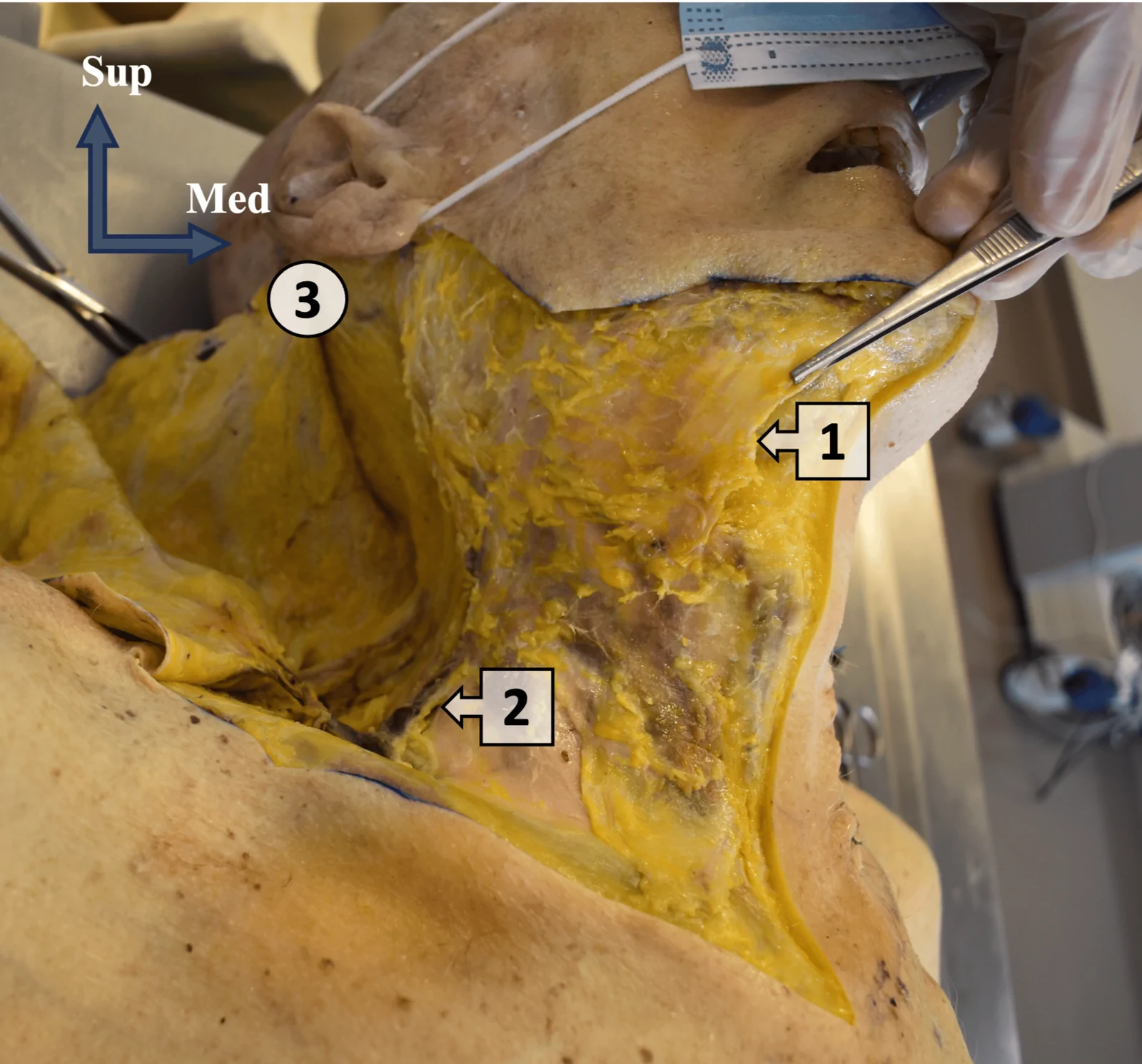
Reflection of skin and subcutaneous tissue. Lateral reflection of the superficial skin flap toward the mastoid process and acromion, exposing the underlying superficial neurovascular plane : platysma muscle (1), external jugular vein (2), and mastoid process (3)

Step two - SCM retraction and exposure of the carotid triangle: following reflection of the platysma, the SCM is identified as enclosed within the investing layer of the superficial cervical fascia. The muscle is progressively mobilized and retracted posterolaterally to expose the deeper space. The omohyoid muscle is then individualized, enclosed in middle cervical fascia, with particular attention directed to its intermediate tendon. The latter crosses anteriorly to the carotid sheath and serves as a reliable anatomical landmark defining the inferior boundary of the carotid triangle proper (distinct from the caudal boundary of the overall dissection field at the clavicle). Following the plane of the omohyoid permits progressive exposure of the carotid sheath while keeping surrounding structures in view (Figures 3, 4).

**Figure 3:**
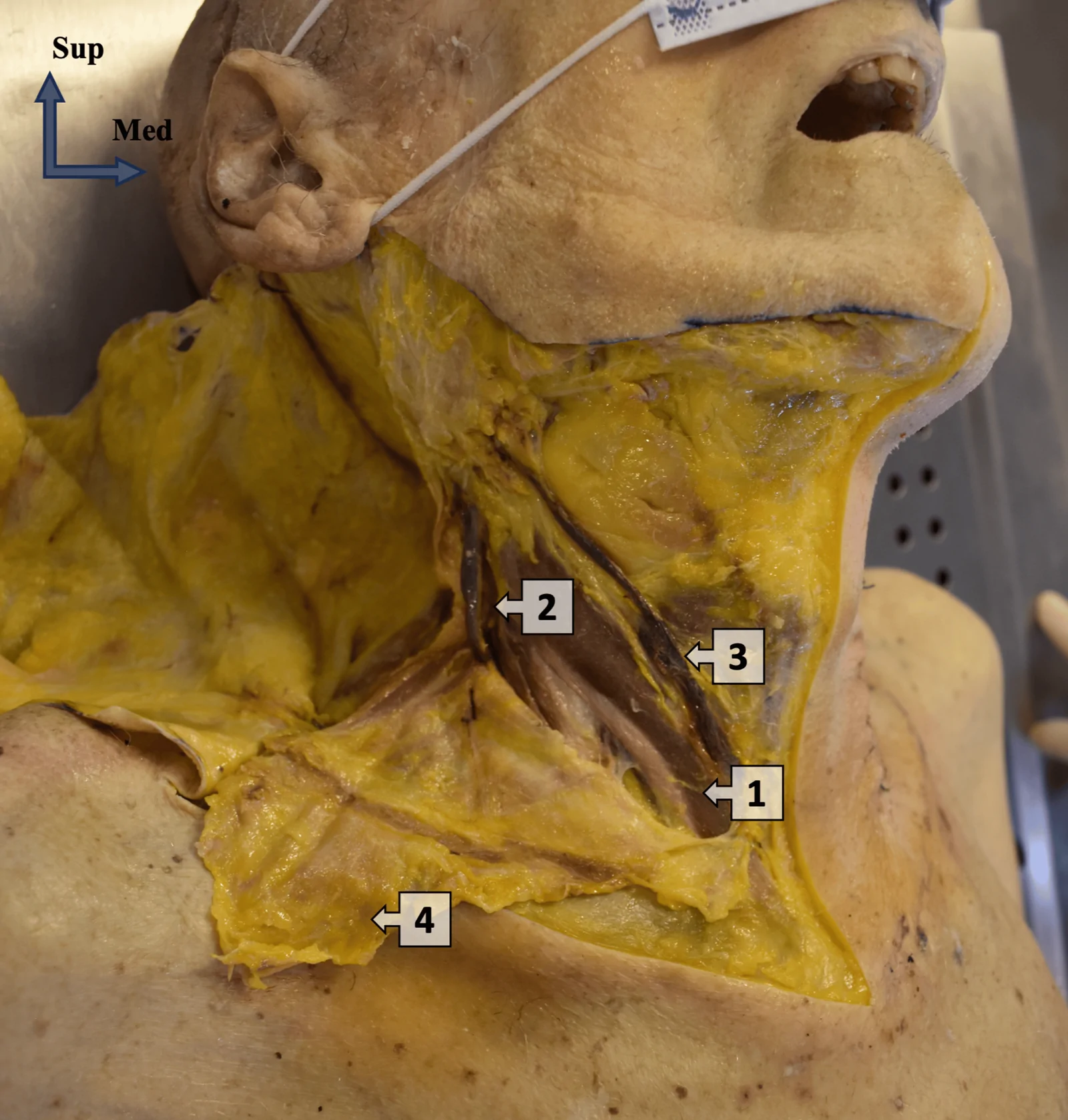
Reflection of the platysma muscle. Sternocleidomastoid muscle (1), external jugular vein (2) crossing the lateral surface of the SCM in an anterosuperior to posteroinferior direction, anterior jugular vein (3), and reflected platysma muscle (4).

**Figure 4:**
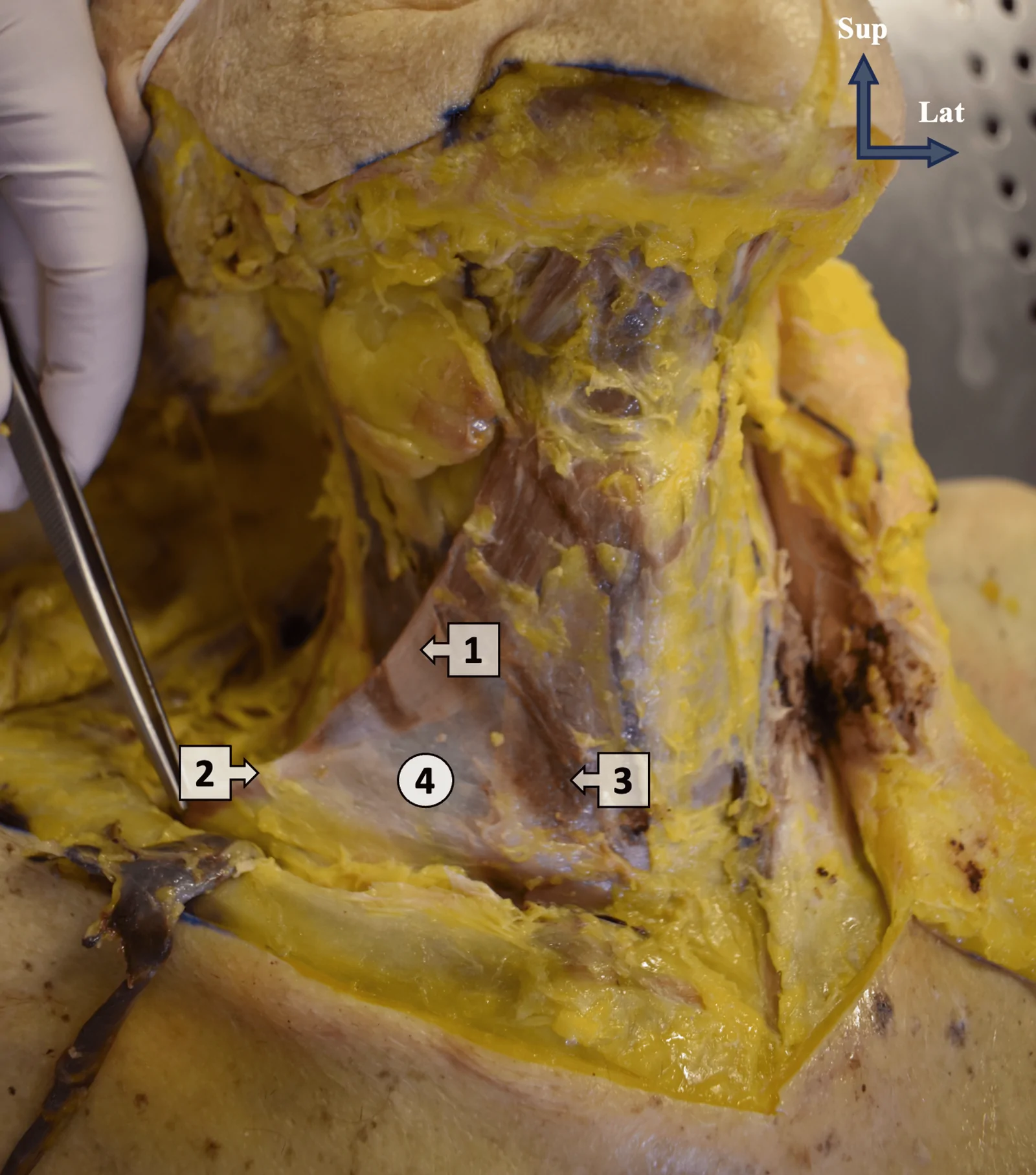
Reflection of the sternocleidomastoid muscle. Deep dissection of the infrahyoid muscular plane and middle cervical fascia : superior belly of the omohyoid muscle (1) and its intermediate tendon (2) posteriorly, inferior portion of the sternohyoid muscle (3) anteriorly, and middle cervical fascia (4).

Step three - internal jugular vein (IJV), venous confluence, and Farabeuf's triangle: Dissection of the jugular lymph nodes and longitudinal opening of the carotid sheath allow systematic identification of the IJV and its anterior tributaries. Deep exposure reveals the thyro-linguo-facial venous trunk (TLFT), formed by the confluence of the facial, lingual, and superior thyroid veins. In contrast, the middle thyroid vein is identified draining directly and independently into the IJV inferior to the main confluence. Alternatively, separate primary drainages of the facial, lingual, or superior thyroid veins into the IJV are noted depending on anatomical variations. Opening the sheath also brings into view the common carotid artery (CCA) medially and the vagus nerve posterolaterally. Finally, mobilization and section of the submandibular gland provides clear visualization of the posterior belly of the digastric muscle, defining the superior boundary of the deep vascular space (Figure 5).

**Figure 5:**
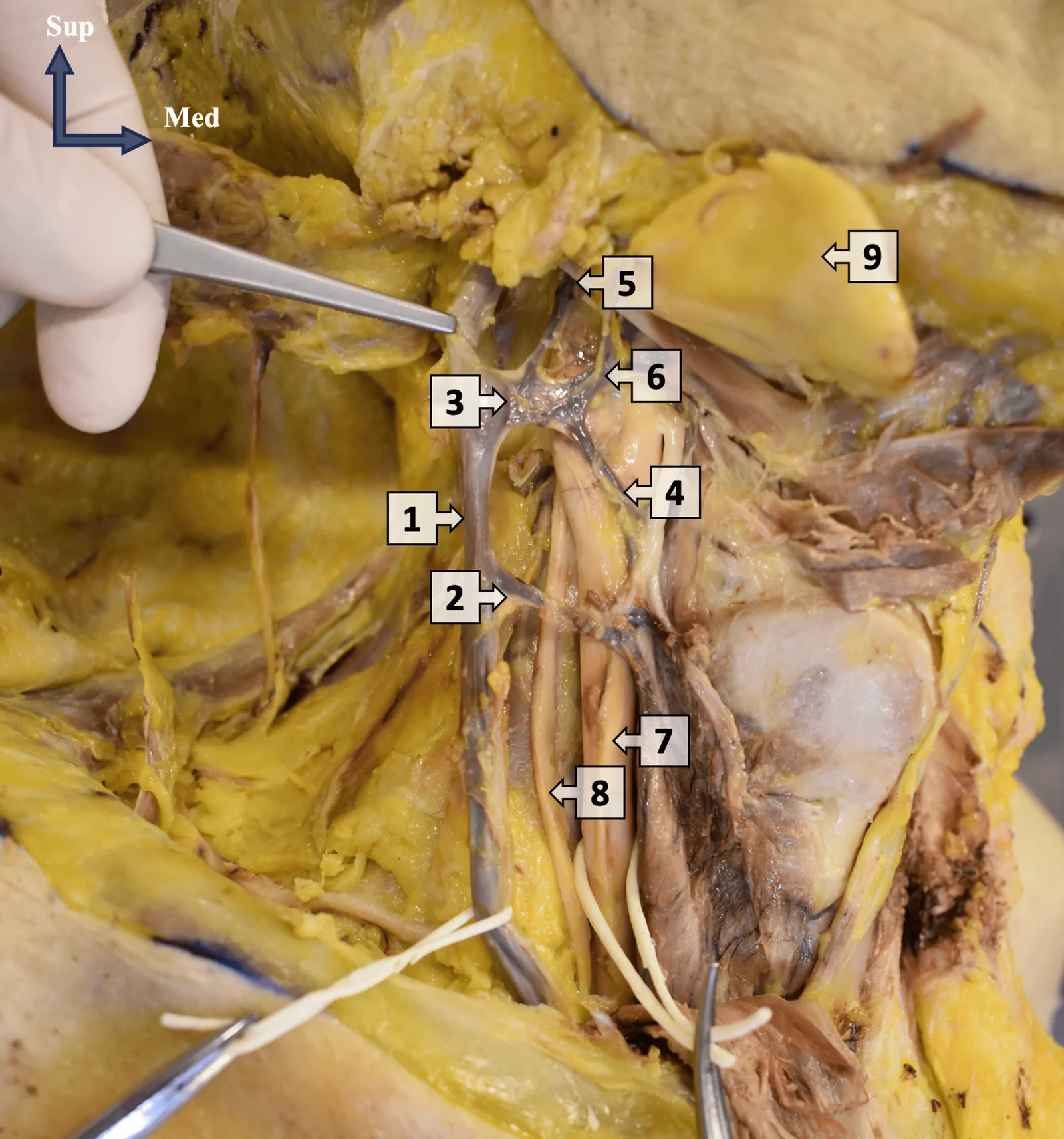
Longitudinal incision of the carotid sheath and exposure of the neurovascular bundle. Full-length opening of the carotid sheath demonstrating its primary contents : Internal Jugular Vein (1), Middle Thyroid Vein (2), Thyro-Linguo-Facial Trunk (3) formed by the confluence of the Superior thyroid Vein (4), facial vein (5), and lingual vein (6), common carotid Artery (7), Vagus Nerve (CN X) (8) superiorly reflected Submandibular Gland (9).

Step four - arterial exposure and carotid sheath isolation: Because the common venous trunk crosses the arterial axis anteriorly, it is transected and retracted along with the middle thyroid vein, permitting lateral mobilization of the IJV. The CCA is then identified medially relative to the laterally situated IJV, while the vagus nerve (CN X) occupies the posterior position within the sheath. This step provides a fundamental surgical guide: before any manipulation of the carotid vessels, the artery, vein, and vagus nerve must be individually identified and verified.

Step five - carotid bifurcation and arterial branches: The CCA is traced superiorly to its bifurcation into the internal carotid artery (ICA) and external carotid artery (ECA). The bifurcation is generally situated at the upper margin of the thyroid cartilage, corresponding to the C3-C4 vertebral level; however, this level remains a variable anatomical landmark rather than an absolute rule. The ICA is recognized by its straight ascending course and the complete absence of cervical collateral branches. Conversely, the ECA is distinguished by its characteristic cervical branching pattern, giving rise to its anterior branches (superior thyroid, lingual, and facial arteries) and posterior branches (occipital, posterior auricular, and ascending pharyngeal arteries) before entering the parotid gland.

At the proximal segment of the ICA, the carotid sinus appears as a localized dilation, while the carotid body is situated within the adventitial intercarotid tissue. Accurate differentiation between the ICA and ECA is essential before vascular cross-clamping, while knowledge of the carotid sinus and carotid body is vital for preventing reflex cardiovascular depression and guiding dissection during paraganglioma resections (Figures 6, 7).

**Figure 6:**
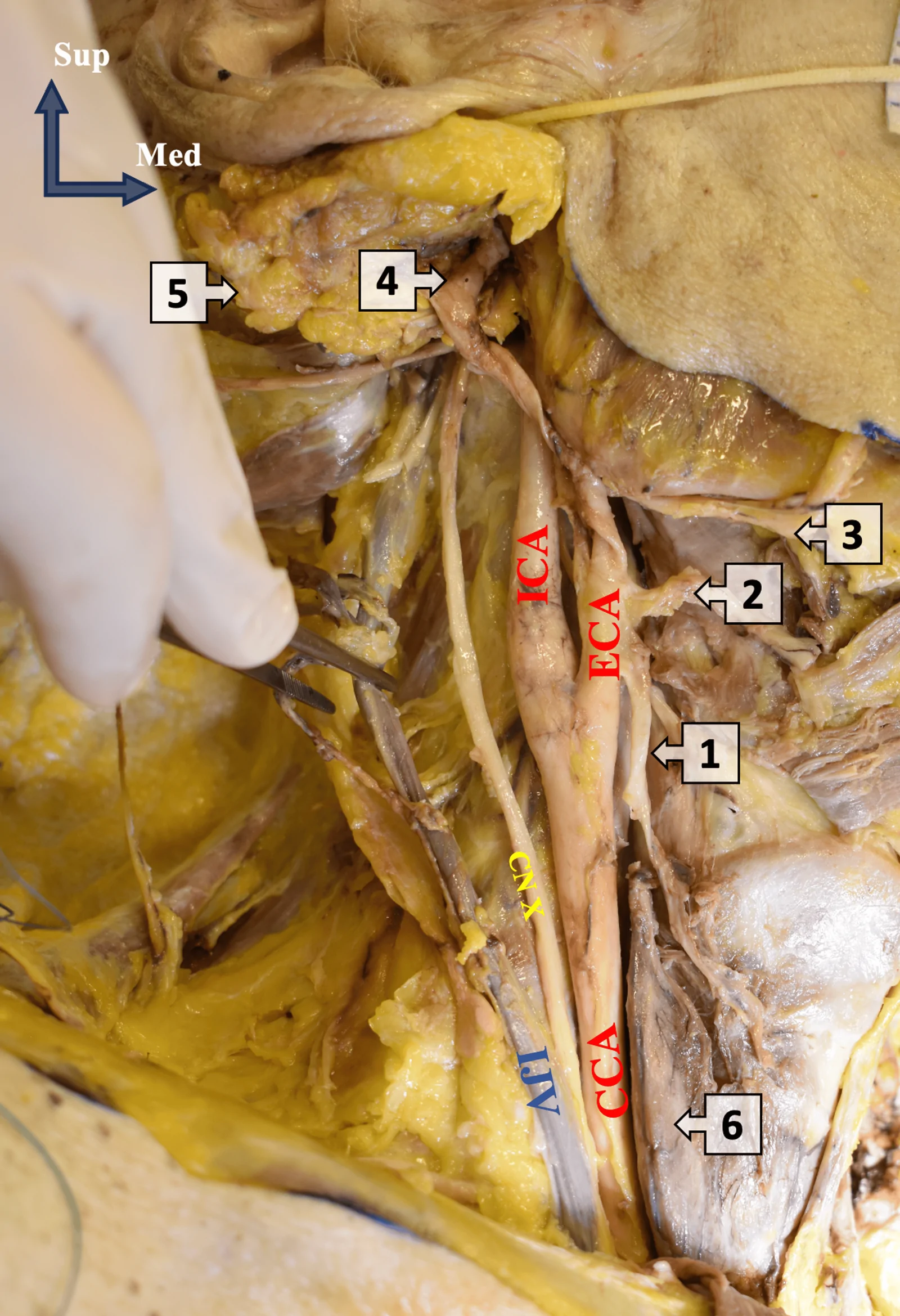
Dissection of the anterior collateral branches of the external carotid artery (ECA). Superior thyroid Artery (1), Lingual Artery (2), facial artery (3), Intra-parotid Segment of the external carotid artery (4), dissected Parotid gland (5), and Thyroid Gland (6).

**Figure 7:**
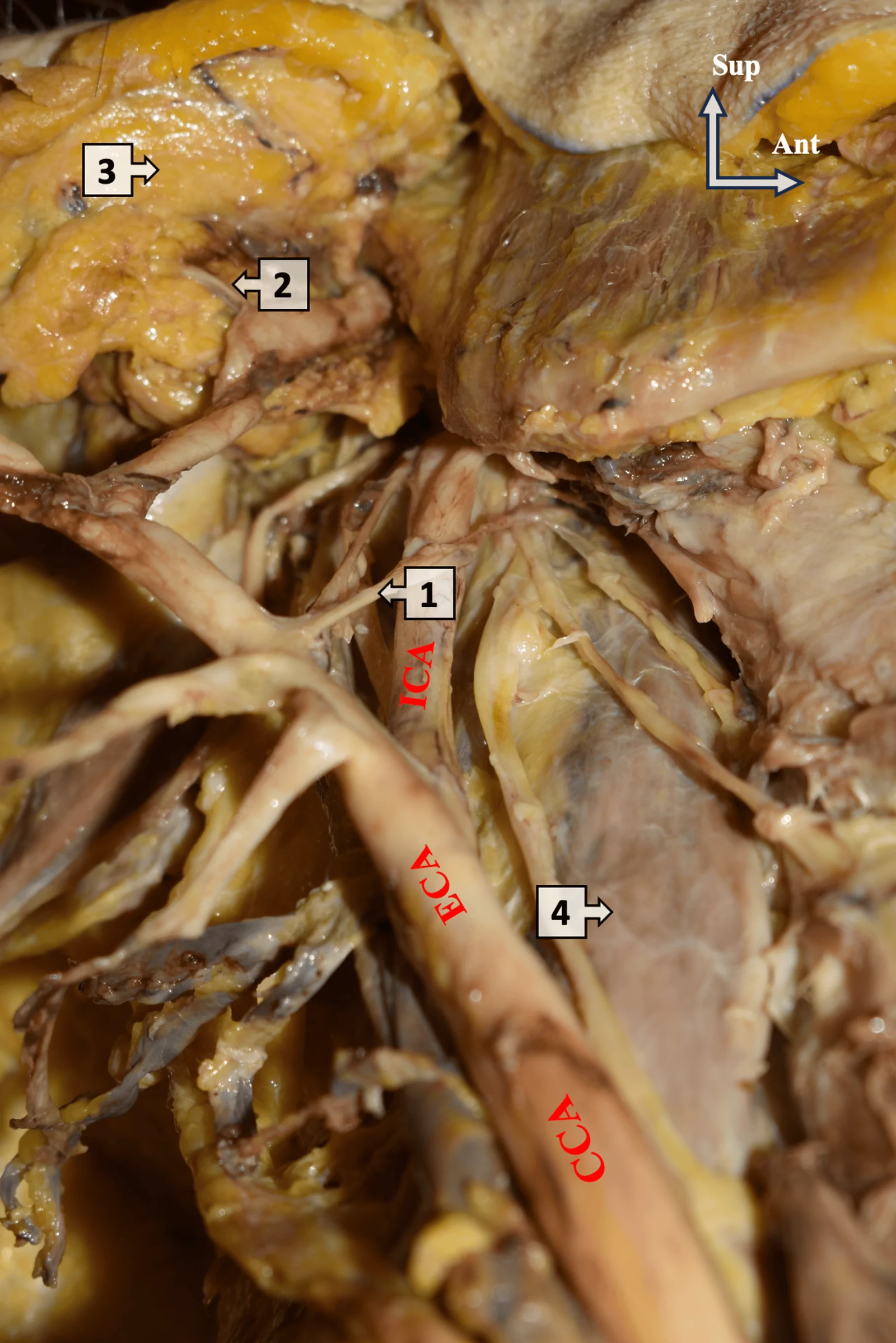
Dissection of the posterior collateral branches of the external carotid artery (ECA). Ascending Pharyngeal Artery (1), Posterior Auricular artery (2), Parotid Gland (3), and Esophagus (4). Note that the occipital artery is not visible in this specific exposure plane.

Step six - identification of adjacent nerves: At the superior border of the region, sectioning of the posterior belly of the digastric and stylohyoid muscles fully exposes the distal course of the hypoglossal nerve (CN XII) as it passes across the lateral aspect of the carotid arteries, extending anteroinferiorly toward the suprahyoid region. Its spatial relationship with the carotid bifurcation and the origin of the occipital artery is documented. Due to its close relationship with the distal carotid axis, CN XII represents a high-risk structure during high cervical exposure and level II neck dissection, where excessive traction or direct trauma leads to ipsilateral tongue dysfunction.

Along the anterior surface of the carotid sheath, the superior root of the ansa cervicalis is isolated and traced inferiorly. This root, composed mainly of C1 fibers, runs temporarily alongside CN XII before descending to innervate the infrahyoid musculature. Within the deep layer of the sheath, the vagus nerve (CN X) is identified in its characteristic posterior position relative to the common and internal carotid arteries and traced toward the jugular foramen (Figures 8, 9). 

**Figure 8:**
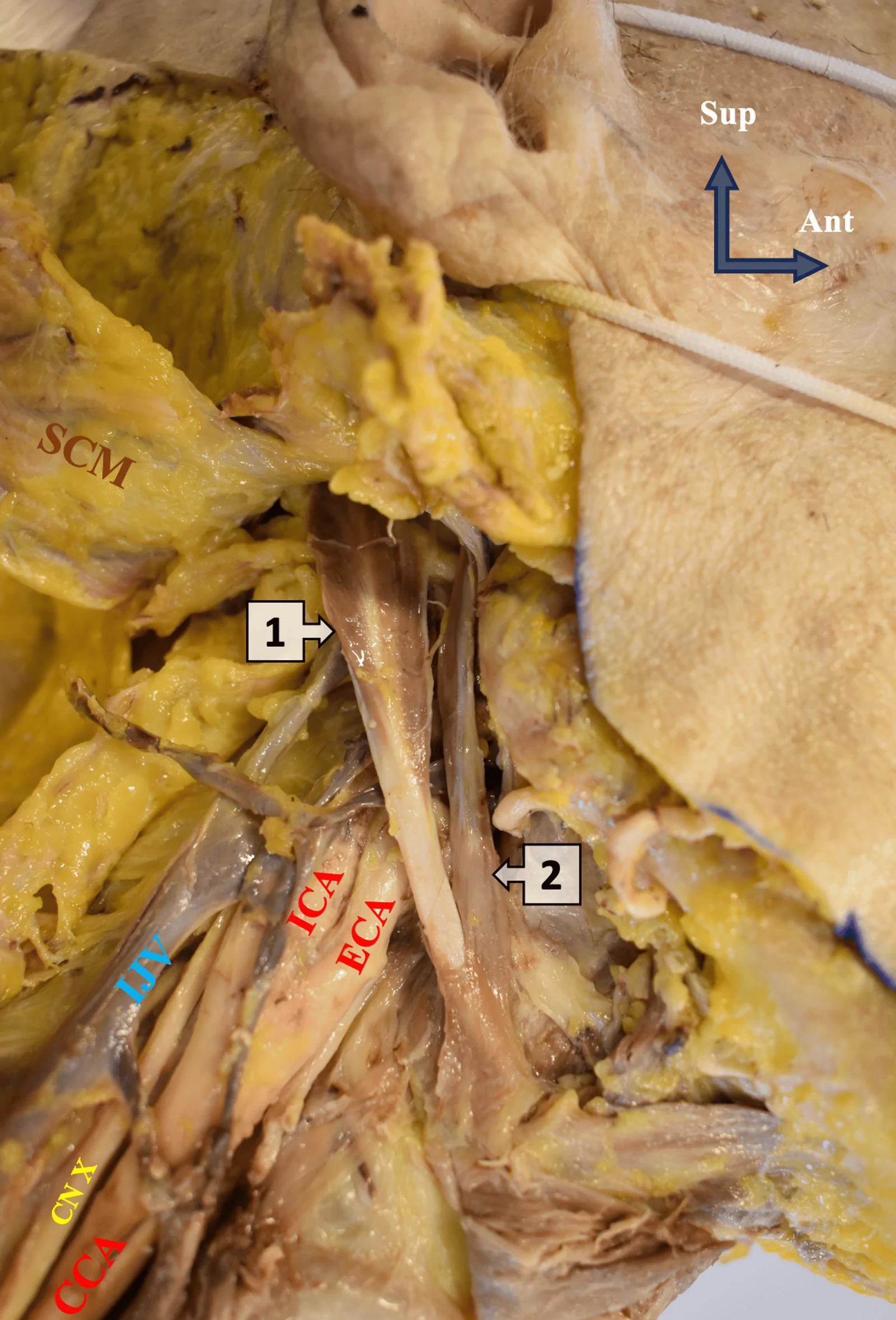
High carotid exposure following submandibular gland resection. Removal of the submandibular gland exposes the superior anatomical boundary of the carotid triangle : posterior belly of the digastric muscle (1), stylohyoid muscle (2)

**Figure 9:**
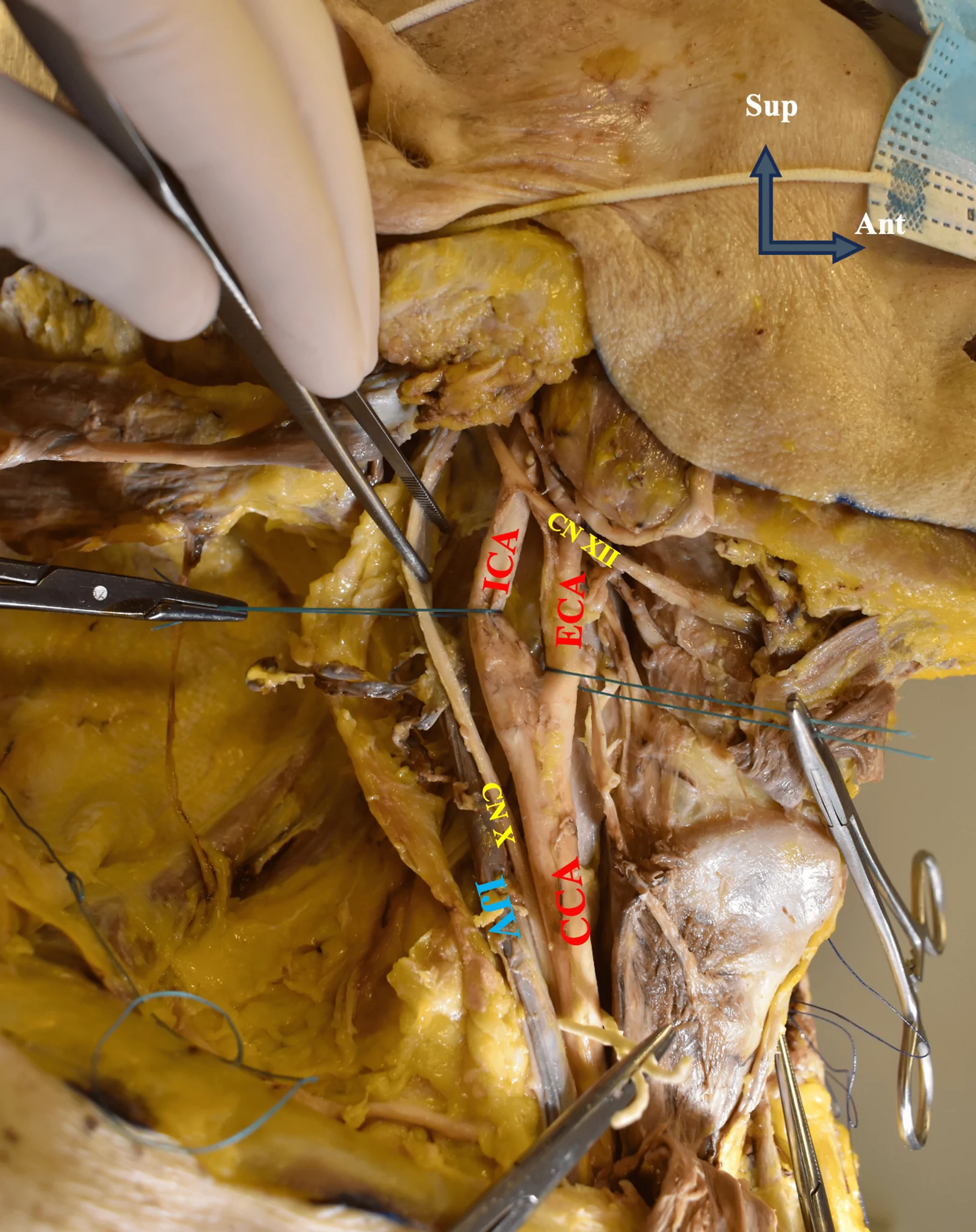
Global view of the neurovascular landmark Course of the hypoglossal nerve (CN XII) and overview of the carotid region.

## Discussion

The clinical and educational value of this step-by-step dissection lies in establishing a precise three-dimensional framework for surgical procedures involving the anterolateral neck. Initial navigation relies on superficial landmarks and fascial planes to guide exposure toward the carotid sheath while preserving superficial neurovascular structures [3]. It is essential to distinguish the broad, quadrilateral carotid region-bounded caudally by the clavicle-from the classic carotid triangle, which is confined inferiorly by the omohyoid muscle [4]. Deep to the investing fascia, Farabeuf's triangle provides an indispensable surgical window during cervical node dissections and vascular control. However, the reliability of this landmark depends heavily on venous architecture; a distinct thyro-linguo-facial trunk is not universally present, and separate drainage of the facial, lingual, or superior thyroid veins into the IJV must be anticipated. The middle thyroid vein represents an independent tributary that requires separate division to mobilize the IJV laterally. Upon opening the carotid sheath, early, individual identification of the common carotid artery, IJV, and vagus nerve (CN X) establishes a safe anatomical baseline before any vascular manipulation [5,6].

Accurate differentiation between the internal carotid artery (ICA) and external carotid artery (ECA) at the bifurcation is paramount before applying cross-clamps. Although the absence of cervical collateral branches remains the most reliable anatomical criterion for identifying the ICA, thorough anatomical inspection is required due to documented variations. The level of carotid bifurcation is itself highly variable, ranging from C2 to C5, which can significantly alter the operative field during high carotid exposures. Furthermore, the origin of the superior thyroid artery frequently deviates, arising directly from the bifurcation or the common carotid artery [7].

Neural structures within the carotid region demand active identification and meticulous protection. The hypoglossal nerve (CN XII) is particularly vulnerable as it crosses the carotid axis and requires gentle mobilization during high cervical exposures. The external branch of the superior laryngeal nerve, coursing close to the superior thyroid pedicle, requires careful preservation to prevent vocal fatigability and loss of high-pitched phonation. Additionally, awareness of specialized vascular structures-the carotid sinus and carotid body-is essential to prevent intraoperative reflex bradycardia and hypotension, and to navigate hypervascular planes during paraganglioma resections [8].

This standardized protocol directly mirrors the operative sequences required in carotid endarterectomy, selective neck dissections (levels II-III), and carotid body tumor resections. The anatomical orientation provided by this protocol is also helpful during anterior cervical spine approaches (such as the Smith-Robinson approach), where the carotid sheath represents the critical lateral boundary of dissection. As a pedagogical tool, it offers a reproducible cadaveric teaching model that enables trainees to master spatial relationships rather than memorizing isolated structures [9].

Technical pearls and pitfalls

Mastering carotid region dissection requires precise technical execution and an awareness of potential anatomical traps [10,11].

Systematic Sheath Verification

Confirm the individual identity and course of the carotid artery, IJV, and vagus nerve before initiating vascular mobilization.

Dynamic Landmark Anticipation

Treat the level of carotid bifurcation and the anterior cervical venous pattern as variable anatomical features rather than fixed assumptions.

Vascular Cross-Clamping Safety

Confirm the absence of collateral branches along the entire exposed cervical length of the ICA before clamping.

Hypoglossal Nerve Preservation

Active early identification of CN XII is necessary during high carotid exposures before applying traction or distal vascular clamps.

Pre-Division Venous Assessment

Map all anterior tributaries entering the IJV before transection, recognizing that the middle thyroid vein drains separately from the main venous confluence.

Limitations

This study has several inherent limitations. First, the protocol is derived from a single cadaveric specimen, which precludes the quantitative assessment of anatomical variations such as carotid bifurcation levels, atypical branching patterns, or venous tortuosity. Second, no morphometric measurements were recorded, which restricts direct metric comparisons across diverse anatomical cohorts. Future multi-specimen studies incorporating rigorous quantitative morphometry are recommended to complement this qualitative dissection roadmap.

## Conclusions

This standardized six-step cadaveric dissection protocol offers a clear, reproducible framework for mastering the complex neurovascular relationships of the carotid region, serving as a practical educational bridge between theoretical anatomy and intraoperative execution across vascular surgery, otolaryngology, and cervical spine approaches. Nevertheless, the qualitative nature of this report-derived from a single cadaveric specimen-represents its primary limitation, as it cannot capture the full spectrum of anatomical variations encountered in clinical practice. Future perspectives should focus on validating this protocol across multi-specimen cohorts to document morphological variants, comparing its educational efficacy against traditional dissection techniques, and integrating these cadaveric steps with 3D radiological imaging and virtual simulation tools to enhance surgical training.
